# RNA-seq expression profiling of rat MCAO model following reperfusion Orexin-A

**DOI:** 10.18632/oncotarget.22995

**Published:** 2017-12-06

**Authors:** Chun-Mei Wang, Yan-You Pan, Ming-Hui Liu, Bao-Hua Cheng, Bo Bai, Jing Chen

**Affiliations:** ^1^ Neurobiology Institute, Jining Medical University, Jining, P.R. China; ^2^ Division of Biomedical Sciences, Warwick Medical School, University of Warwick, Coventry, UK

**Keywords:** Orexin-A, ischemia-reperfusion injury, RNA sequencing, differential gene expression, gene network

## Abstract

Orexin-A is a neuropeptide with potent neuroprotective activity towards cerebral ischemia-reperfusion (I/R) injury, but few studies have attempted to elucidate the mechanism. Herein, we performed global gene expression profiling of the hippocampus following reperfusion with Orexin-A using RNA sequencing (RNA-seq). RNA-seq identified 649 differentially expressed genes (DEGs) in the Orexin-A group compared with saline controls (I/R group), of which 149 were up-regulated and 500 were down-regulated. DEGs were confirmed using qRT-PCR, their molecular functions, biological processes and molecular components were explored using Gene Ontology (GO) analysis and 206 KEGG pathways were associated with Orexin-A treatment. MAPK, chemokine and calcium signalling pathways were mainly responsible for the neuroprotective effects of Orexin-A. Hspb1, Igf2 and Ptk2b were selected for functional interaction analysis by GeneMANIA. The results suggest that Orexin-A modifies gene expression in the hippocampus, leading to neuroprotection from I/R injury. The study provides a basis for future elucidation of the molecular mechanisms underlying Orexin-A.

## INTRODUCTION

Orexin-A is a novel neuropeptide involved in the regulation of feeding behaviour [1, 2], energy metabolism [3], hormone secretion [4], the sleep-wake cycle [5] and anaesthesia [6]. Orexin-A plays a pivotal role in neurotrauma [7], Parkinson's disease [8], narcolepsy [9], vascular diseases [10] and brain tumours [11, 12]. In addition, Orexin-A protects against ischemia-reperfusion (I/R) injury by reducing gastric lesions through increasing HO-2 expression and decreasing HO-1 expression [13]. After intestinal I/R injury, hypothalamus Orexin-A levels gradually decrease from 30 to 150 min of reperfusion, and then recover slowly [14]. Orexin-A levels change during hepatic reperfusion, indicating involvement in the regulation of liver injury induced by this process [15].

Similarly, Orexin-A has a prominent neuroprotective effect on cerebral I/R injury. Kitamura et al. found that the number of neurons expressing Orexin-A was significantly lower in the non-ischemic side than the ischemic side [16]. Additionally, they found Orexin-A pre-treatment significantly reduced the brain infarction volume induced by reperfusion injury, ameliorated neurologic deficit scores and reduced the infarction volume after rat cerebral I/R injury. The neuroprotective effect of Orexin-A was shown to be mediated by an increase in hypoxia-inducible factor-1 (HIF-1α) activity [17], and our lab demonstrated that Orexin-A protects SHY-5Y cells against oxidative stress by activating the PI3K/AKT signalling pathway.

Few studies have investigated the neuroprotective effects of Orexin-A in I/R injury. Therefore, in the present study we performed global gene expression profiling in the hippocampus after reperfusion with Orexin-A by RNA sequencing (RNA-seq). We identified differentially expressed genes (DEGs) and signalling pathways putatively associated with Orexin-A reperfusion. The findings provide a basis for further elucidation of the molecular mechanisms underlying the neuroprotective effects of Orexin-A.

## RESULTS

### Orexin-A decreases the infarct volume after cerebral I/R injury

**Figure 1:**
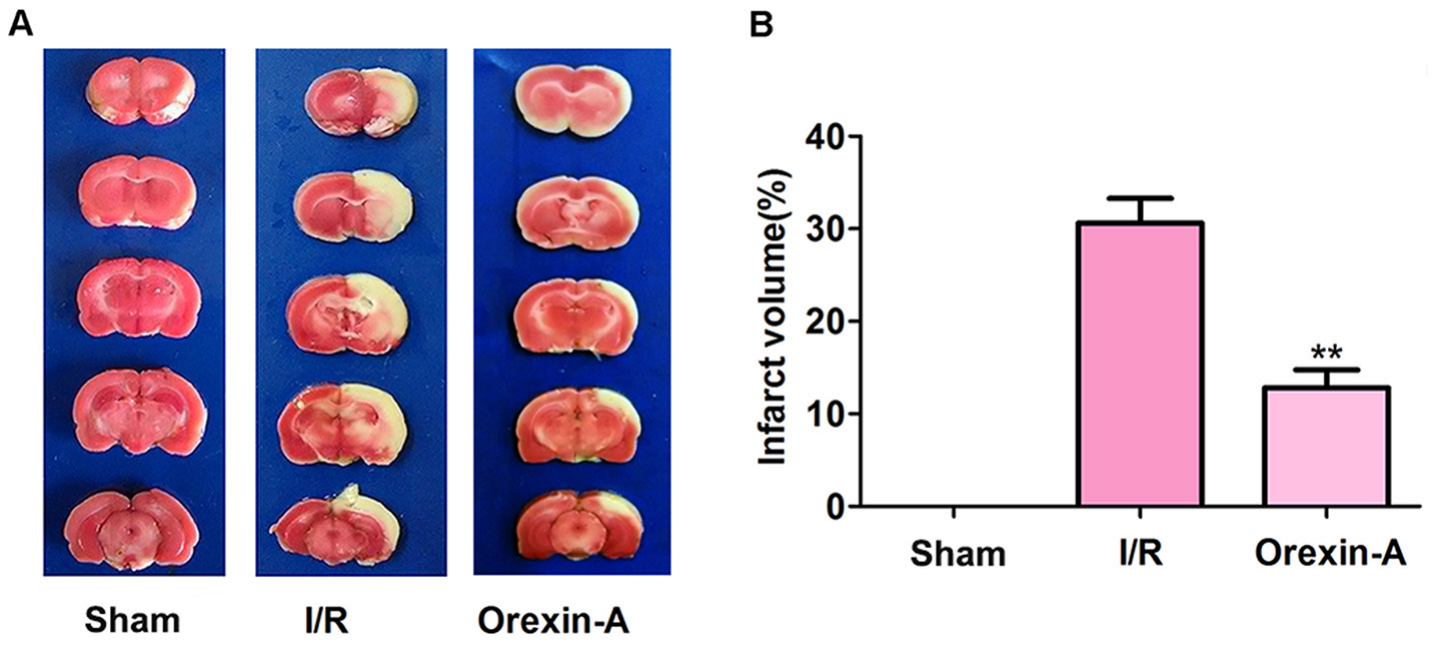
Orexin-A decreases the cerebral infarction volume following middle cerebral artery occlusion (MCAO) in rats **(A)** Representative images from sham and MCAO groups treated with or without Orexin-A after a 24 h reperfusion using TTC staining. Brain sections from the sham group are red, and infarction volumes are not visible. Infarction volumes in the I/R group are white and obviously increased in size compared with the sham group. Reperfusion of Orexin-A significantly decreases the infarction volume compared with the I/R group. **(B)** Percentage of cerebral infarction volumes to total brain volumes. Columns represent infarction volumes as a percentage of total volumes. Data are expressed as mean ± SD (n = 6). ^**^p < 0.01 vs. the I/R group.

Infarction volumes in model rats were measured using triphenyltetrazolium chloride (TTC) staining. As shown in Figure 1, normal tissues were stained red, while infarction areas were stained white. No infarction area was identified in the sham group, while the I/R group displayed a significantly higher rate of infarction (30.620% ± 2.665%) than the sham group. In addition, the Orexin-A group displayed a rate of infarction that was 12.497% ± 1.912% lower than that displayed by the I/R group (^**^p < 0.01), indicating that pre-treatment with Orexin-A significantly decreased the infarction area, and Orexin-A therefore exhibited a clear neuroprotective effect on I/R injury.

### Global analysis of RNA-seq data

**Figure 2:**
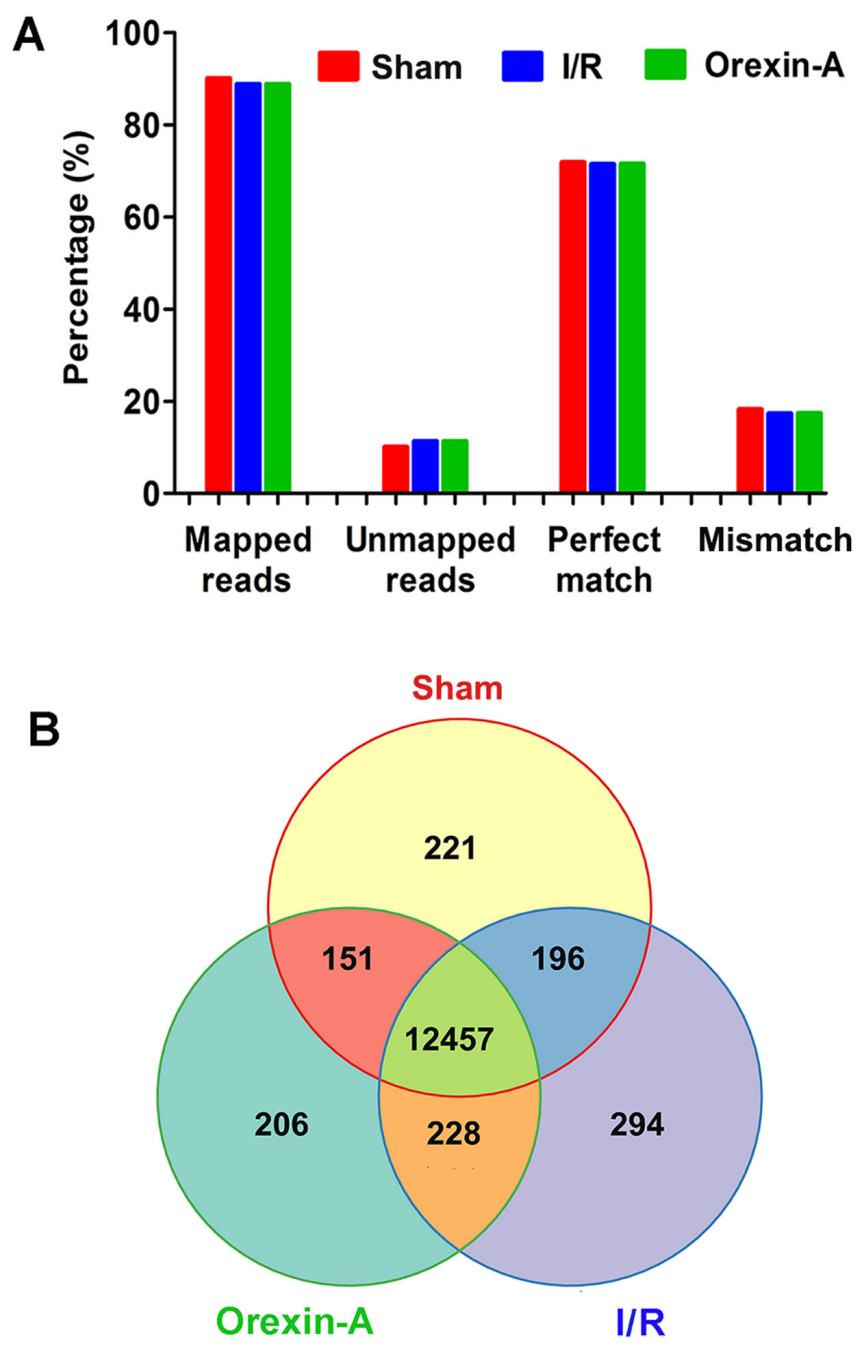
Analysis of global gene expression using RNA-seq data **(A)** Summary of RNA-seq reads mapped. The y-axis represents the percentage of mapped reads or genes. **(B)** Venn diagrams showing the number of commonly and uniquely expressed genes among the three treatment groups.

Clean reads from RNA-seq were filtered, and 14,199,598, 13,499,205 and 13,116,887 total reads were acquired in the sham, I/R and Orexin-A groups, respectively. Analysis of global gene expression is shown in Figure 2. About 89% of reads were successfully mapped, 11% were unmapped, 71% were perfectly matched with reference sequences and 17-18% were mismatched (Figure 2A). Figure 2B shows the number of common and unique genes in the sham, I/R and Orexin-A groups. A total of 12,457 genes were found to be commonly expressed in all three groups, and 221 genes were specific to the sham sample, 294 were unique to the I/R group and 206 were expressed only in the Orexin-A group.

### Screening of DEGs

**Figure 3:**
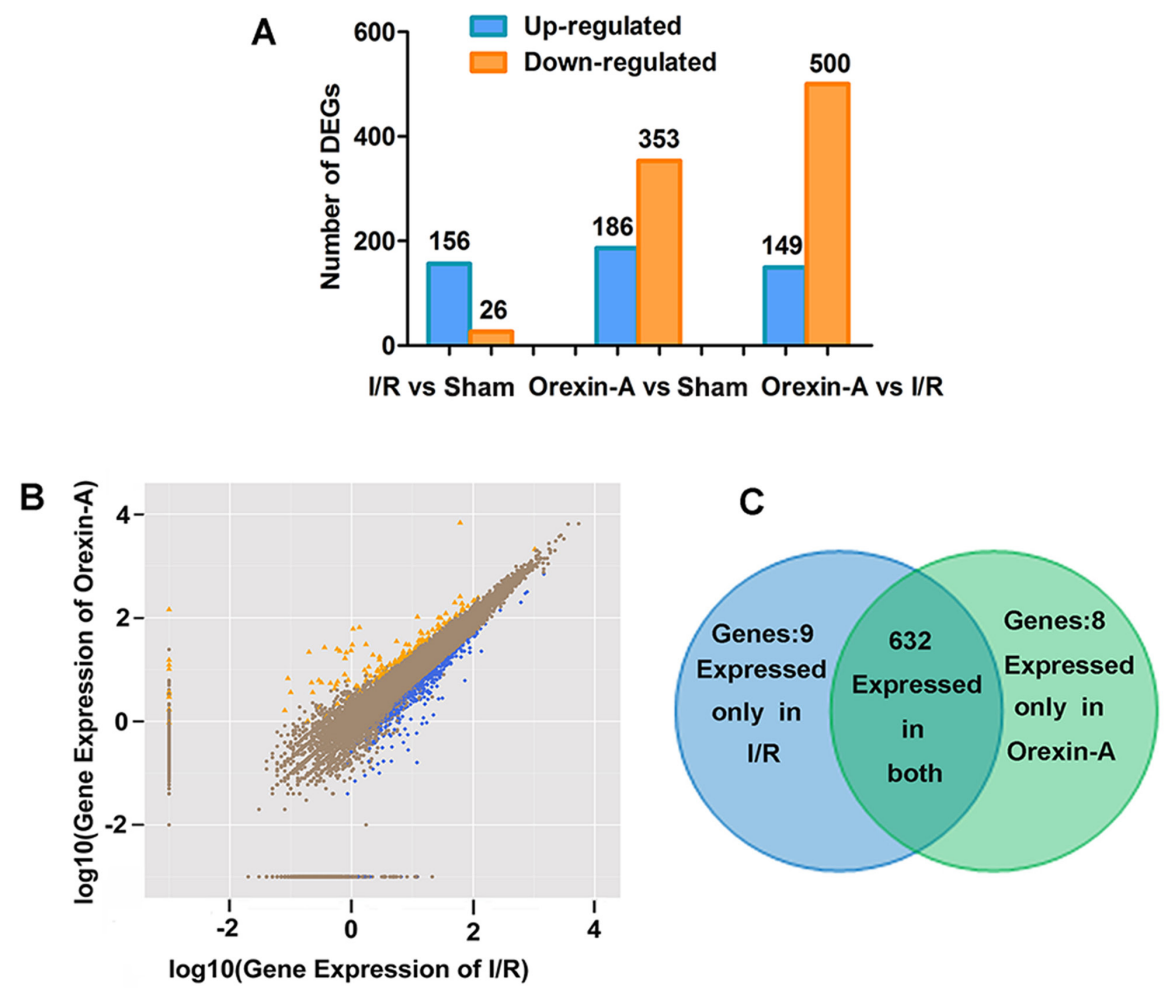
Diagram of differentially expressed genes **(A)** Number of up- and down-regulated genes in each group. **(B)** Scatter plot displaying differentially expressed genes between the I/R and Orexin-A groups. The x- and y-axes show the fold change (log2 ratio) in gene expression. Yellow points represent up-regulated genes with a fold change less than 1 and *p* < 0.05; blue points denote down-regulated genes with a fold change greater than 1 and *p* < 0.05. Brown points represent genes with no significant differences. **(C)** Venn diagrams showing the number of commonly and uniquely differentially expressed genes between the I/R and Orexin-A groups. Nine genes were expressed only in the I/R group, and eight genes were expressed only after reperfusion with Orexin-A.

Genes were considered differentially expressed when FDR ≤0.001 and |log2 ratio| >1. As shown in Figure 3A, 156 genes were up-regulated and 26 genes were down-regulated in the sham group compared with the I/R group. After reperfusion with Orexin-A, 649 genes were differentially expressed compared with the I/R group, of which 149 were up-regulated and 500 were down-regulated. In addition, there were 186 up-regulated genes and 353 down-regulated genes in the Orexin-A group compared with the sham group. Figure 3B shows the number of DEGs and the fold change in expression between the Orexin-A and I/R groups. The absolute fold change (log2 ratio) ranged from 1 to 17.13. Figure 3C shows the expression levels of DEGs in the I/R and Orexin-A groups. Nine genes (Glycam1, Hfe2, Hmgn5, Lect1, LOC100911576, Mybl2, Scgb1c1, Tmem72 and Tmem27) not expressed in the I/R group were significantly up-regulated after reperfusion with Orexin-A. Eight genes (Hmgn5b, LOC100362172, Upk1b, Tmprss5, Msln, Pcdha1, Pcdh11x and Hdx) expressed in the I/R group were not expressed at all after reperfusion with Orexin-A. Additionally, 632 genes were detected in both groups. A selection of DEGs between the Orexin-A and I/R groups is listed in Table 1. These DEGs are likely to be involved in the neuroprotective effects of Orexin-A, and were further investigated.

**Table 1:** List of 20 differentially expressed genes in Orexin-A and I/R groups

**Up-regulated genes**			
**Gene symbol**	**Description**	**Log2 ratio**	***p*-value**
Scgb1c1	secretoglobin, family 1C, member 1	13.8946	1.99E-08
Hmgn5	high mobility group nucleosome binding domain 5	13.5781	7.09E-29
Tmem72	transmembrane protein 72	13.3148	2.53E-41
Tmem27	transmembrane protein 27	11.8455	9.26E-06
Lect1	leukocyte cell derived chemotaxin 1	11.7313	2.37E-06
Hfe2	hemochromatosis type 2	10.7228	7.17E-05
Mybl2	myeloblastosis oncogene-like 2	9.8765	7.17E-05
Ttr	transthyretin	6.7934	1.20E-06
Psca	prostate stem cell antigen	5.1408	4.18E-10
Cdh3	cadherin 3	4.6621	1.64E-19
Fibcd1	fibrinogen C domain containing 1	1.4395	2.14E-73
**Down-regulated genes**			
**Gene symbol**	**Description**	**Log2 ratio**	***p*-value**
LOC100362172	LRRGT00112-like	-12.6795	1.27E-05
Upk1b	uroplakin 1B	-10.9729	5.18E-05
Tmprss5	transmembrane protease, serine 5	-10.7566	1.27E-05
Pcdha1	protocadherin alpha 1	-10.3106	1.11E-08
Pcdh11x	protocadherin 11 X-linked	-9.7813	2.56E-05
Hdx	highly divergent homeobox	-9.7813	5.18E-05
Shox2	short stature homeobox 2	-6.2204	1.82E-21
Bcl3	B-cell CLL/lymphoma 3	-4.9307	4.51E-16
Aspg	asparaginase	-4.8379	4.25E-08
Socs3	suppressor of cytokine signalling 3	-4.0020	1.20E-11
Hspb1	heat shock protein B1	-2.6018	1.48E-58
Slc24a2	solute carrier family 24, member 2	-1.20343	1.64E-05

### qRT-PCR validation

**Figure 4:**
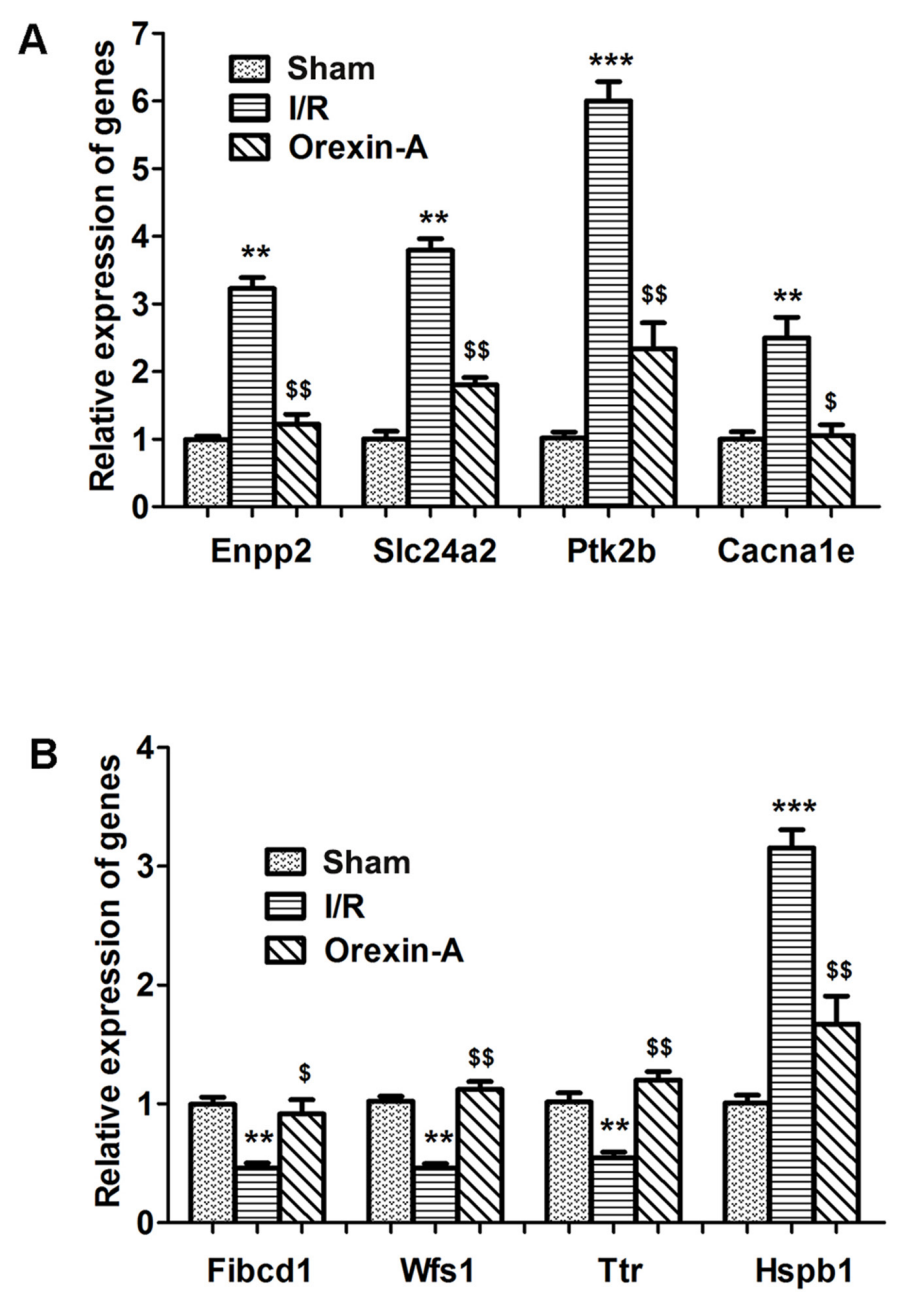
Validation of a selection of differentially expressed genes using qRT-PCR **(A-B)** Fives genes were up-regulated and three were down-regulated in the I/R group compared with the sham group. Treatment with Orexin-A reversed these expression changes. ^**^p < 0.01, ^***^p < 0.001 vs. sham group; ^$^*p* < 0.05, ^$$^*p* < 0.01 vs. I/Rgroup.

qRT-PCR was performed to validate selected DEGs identified by RNA-seq. The relative expression levels of eight DEGs were determined using a LightCycler 480 (LC480). The sequences of primers were listed in Table 2. As shown in Figure 4, the relative expression levels determined by qRT-PCR were in good agreement with the RNA-seq data. Fives genes (Enpp2, Slc24a2, Pk2b, Cacna1e and Hspb1) were up-regulated in the I/R group compared with the sham group (^**^p < 0.01, ^***^p < 0.001), and all were down-regulated following pre-treatment with Orexin-A compared with the I/R group (^$^*p* < 0.05, ^$$^*p* < 0.01). Three genes (Fibcd1, Wfs1 and Ttr) were down-regulated at 24 h after reperfusion compared with the sham group (^**^p < 0.01), whereas Orexin-A reperfusion clearly increased their expression compared with the I/R group (^$^*p* < 0.05, ^$$^*p* < 0.01). The results confirmed the reliability of the RNA-seq data and further indicated the involvement of the identified DEGs in the neuroprotective effects of Orexin-A.

**Table 2:** Sequences of primers used for amplification

Primer name	Forward sequence (5’-3’)	Reverse sequence (5’-3’)
β-actin Enpp2	tggaatcctgtggcatccatgaa cccttcagtccgagtttgacc	taaaacgcagctcagtaacagtccg gccgtccatacaggagatgt
Slc24a2 Ptk2b	ccgaggaagatgatgaccag gccgagttcaagcagatcag	tccagagagggaacacgatg aggtcttgacggccacatta
Cacna1e Hspb1	gcccttaaagctcgtgtcag acgaagaaaggcaggatgaa	gcattggaagacagtcagca gacagggaagaggacaccag
Fibcd1	ggtgtgggcttgttctctgt	gttctctgaatggtcgctgtc
Wfs1	cagccgagaagggacagata	ggcaagtcgcaggtagtgtt
Ttr	ctgctggaaagcctggaa	cactgctctgctcctcttca

### GO annotation of DEGs

**Figure 5:**
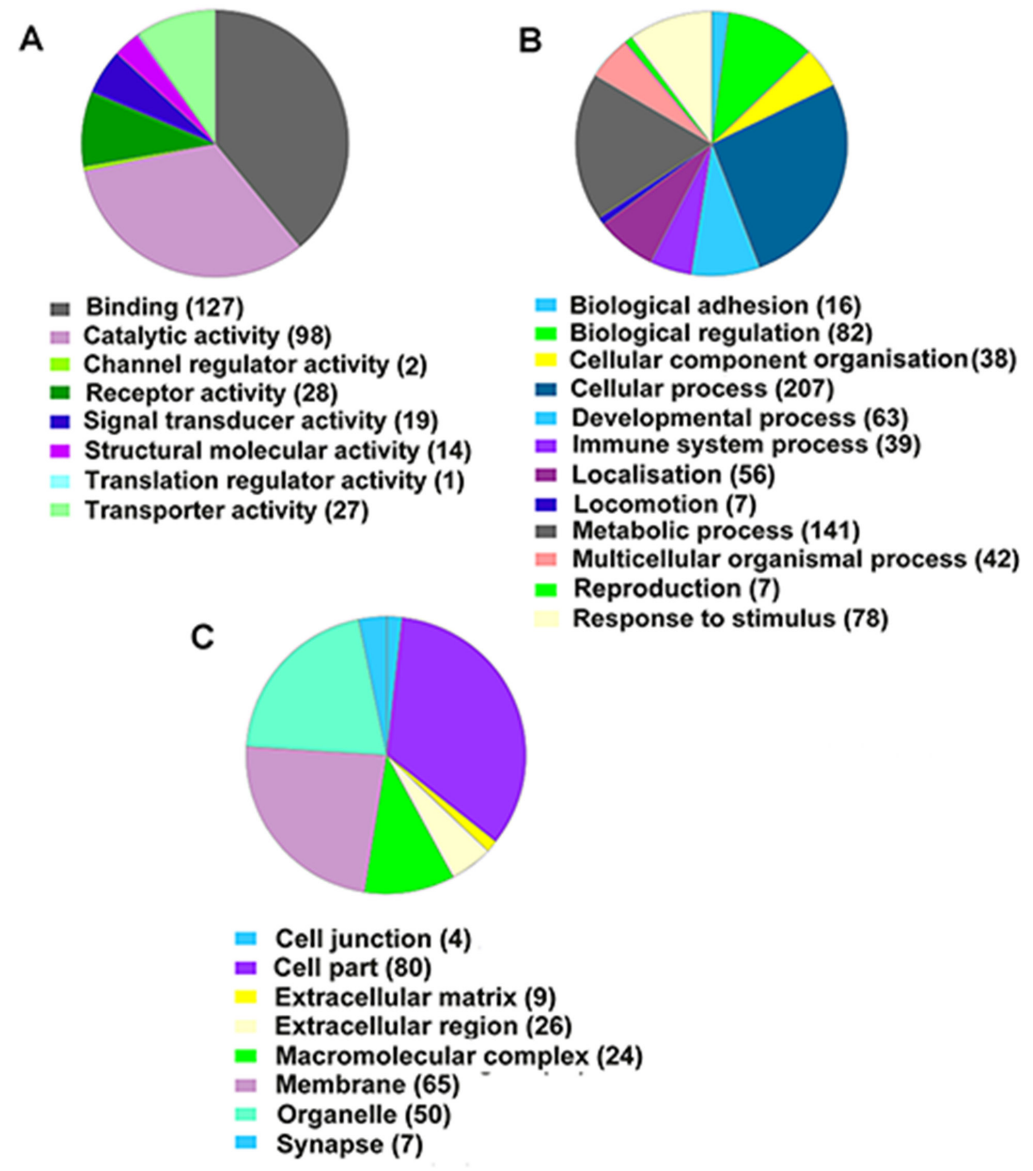
PANTHER classification analysis of differentially expressed genes between I/R and Orexin-A groups **(A)** Molecular function categories. **(B)** Biological process categories. **(C)** Cellular component categories.

GO annotation was used to further investigate the functions of the identified DEGs. As shown in Figure 5, DEGs were annotated into three categories: molecular function, biological process and cellular component. The molecular function category was divided into eight main subcategories, and the top four were binding (GO:0005488), catalytic activity (GO:0003824), receptor activity (GO:0004872) and transporter activity (GO:0005215; Figure 5A). The top hit in the biological process category was cellular process (GO:0009987), metabolic process (GO:0044238), biological regulation (GO:0065007) and response to stimulus (GO:0050896) were also well represented (Figure 5B). Cell part (GO:0044464), membrane (GO:0016020), organelle (GO:0043226) and macromolecular complex (GO:0032991) were the main cellular component subcategories (Figure 5C). A selection of DEGs classified into different subcategories is listed in Table 3.

**Table 3:** GO classification of differentially expressed genes

**Biological process**	
**GO Term**	**Symbol**
GO:0009987: cellular process	Arhgap5, Cobl, Ube2l6, Aqp4, Nrxn1, Inpp5f, Fmr1, Casq2, Capza1, Fras1, Upp1, Clock, Enpp2, Dis3, Aspg;
GO:0044238: metabolic process	Klk8, Cyp2d4, Eif1a, Cfap52, Plcxd3, Lnpep, Eri1, Prkab2, Sult1d1, Gnpda2, Hmgn5, Cdkl1, Met, Pcmtd1, Dbp;
GO:0065007: biological regulation	Oas1i, Gabrb2, Slc17a6, Cd38, Gdf10, Zic1, Taok1, Sostdc1, Dnm1l, Jak2, Bdnf, Otx2, Timp1, Kcne2, Cd44;
GO:0050896: response to stimulus	Klk8, Stc1, Grik2, Gabra1, Ago4, Shox2, Ucp2, Cdc73, Stat3, Necab2, Eltd1, Prkacb, Crem, Shc3, Lcn2;
GO:0032502: developmental process	Gabrb3, Fnbp1l, Ntrk1, Megf10, Gfap, Kdr, Rfx3, Usp13, Igfbp2, G2e3, Ptgs2, Lect1, Ntng1, Hook1, Bmpr2
**Cellular component**	
**GO Term**	**Symbol**
GO:0005623: cell	Ubn2, Bdnf, Jak2, Prkg2, Gad2, Unc5d, Msx1, Wfs1, Prlr, Rb1cc1, Adra2a, Pdp1, PVR, Car6, Scn3a;
GO:0044464: cell part	lkbh7, Fhod3, Zmat4, Tubb6, Smarcad1, Parm1, Negr1, Ptprt, Camk2d, Doc2b, Vgf, Nexn, Bcl3, Gabra3, Pak3;
GO:0043226: organelle	Rbbp8, Hspa4l, Map3k2, Aqp1, Trib1, Psme4, Guf1, Scn1a, Pds5a, Adamts1, Kitlg, Dnase2, Tmbim1, Akap12, Stc1;
GO:0044425: membrane part	Synpr, Aqp4, Nrxn1, Ap5m1, Shc4, Fras1, Stxbp6, Clic6, Slc6a20, Il13ra1, Fcgr3a, Itm2a, Pqlc1, Bst2, Met
**Molecular function**	
**GO Term**	**Symbol**
GO:0005488: binding	Wipf1, Necab1, Grin3a, Mmp9, Kcnj3, Spta1, Wnt9b, Zfp483, Usp32, Dgkb, myd1, Clk1, Atf3, Fibcd1, Atf2;
GO:0003824: catalytic activity	Ace, Mtif2, Trib1, Guf1, Ube3a, Dnase2, Far1, Cdk18, Clybl, Ddx3, March7, Ascc3, Spint2, Ntrk1, Papd4;
GO:0060089: molecular transducer activity	Epha3, S1pr3, Hif1a, Gpr63, Cd3e, Clock, Map3k2, Acvr2a, Bmpr2, Opcml, Il13ra1, Bag4, Mier1, Plcxd3, Bst2;
GO:0005215: transporter activity	Kpna3, Slc24a5, Aqp4, Grin2b, Gabrb2, Gria4, Folr1, Grin3a, Kcnj3, Vldlr, Aqp1, Clic6, Cacnb4, Trpc6, Hcn4

**Figure 6:**
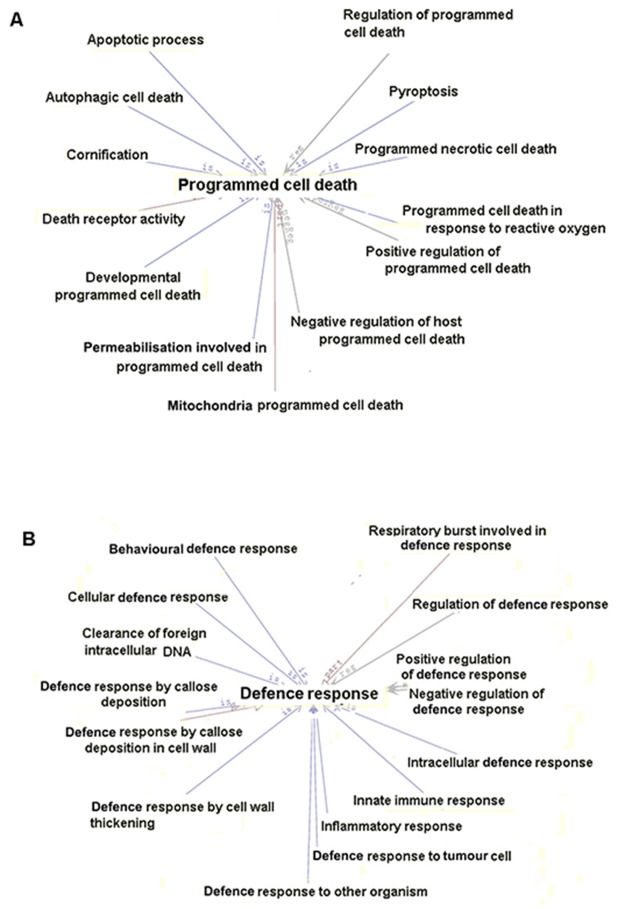
Further analysis of two selected biological process subcategories **(A)** Thirteen subcategories associated with programmed cell death. **(B)** Fifteen subcategories related to defence responses.

We chose two subclasses for further exploration using AmiGO 2 (Figure 6). DEGs related to programmed cell death could be involved in autophagic cell death, cornification, pyroptosis, mitochondria programmed cell death and death receptor activity. DEGs associated with defence responses may play an important role in the cellular defence response, the innate immune response, the inflammatory response, clearance of foreign intracellular DNA and the defence response to tumour cells. Subcategorising DEGs could help us to better understand their potential roles in Orexin-A reperfusion.

### Pathway analysis of DEGs

**Figure 7:**
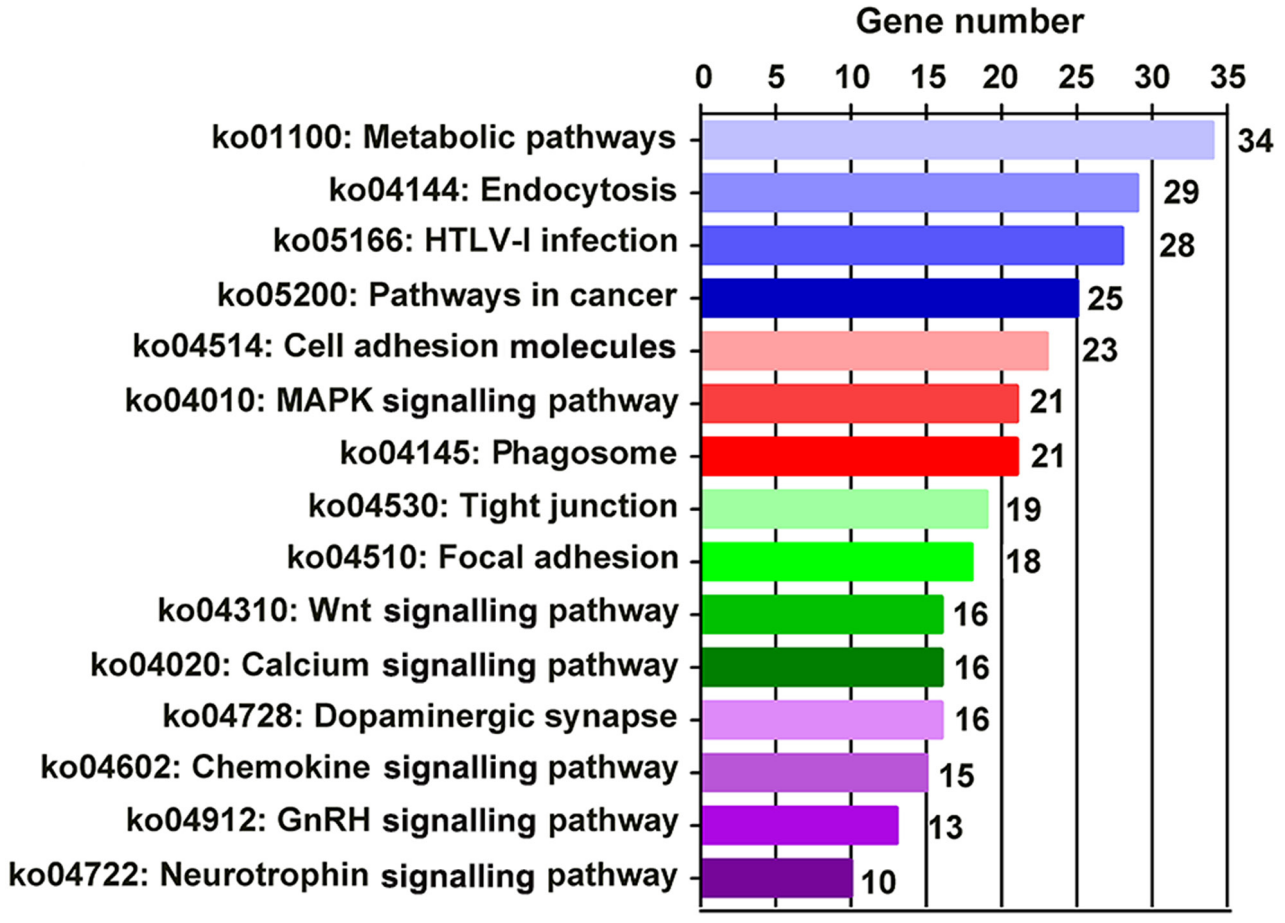
Top 15 enriched pathways between I/R and Orexin-A groups

KEGG pathway analysis was performed to determine the biological pathways associated with DEGs. Among the 206 identified pathways in the Orexin-A group compared with the I/R group, the top 15 were Metabolic pathways (ko01100; 34 DEGs), Endocytosis (ko04144; 29 DEGs), HTLV-I infection (ko05166; 28 DEGs), Pathways in cancer (ko05200; 25 DEGs), Cell adhesion molecules (ko04514; 23 DEGs), Mitogen-activated protein kinase (MAPK) signalling pathway (ko04010; 21 DEGs), Phagosome (ko04145; 21 DEGs), Tight junction (ko04530; 19 DEGs), Focal adhesion (ko04510; 18 DEGs), Wnt signalling pathway (ko04310; 16 DEGs), Calcium signalling pathway (ko04020; 16 DEGs), Dopaminergic synapse (ko04728; 16 DEGs), Chemokine signalling pathway (ko04062; 15 DEGs), GnRH signalling pathway (ko04912; 13 DEGs) and Neurotrophin signalling pathway (10 DEGs; Figure 7 and Table 4). These signalling pathways likely play important roles in a various manner in the neuroprotective effects of Orexin-A.

**Table 4:** KEGG pathway classification of differentially expressed genes

Pathway	Gene symbol
ko01100: Metabolic pathways	Lect1; Ttr; Kl; Pla2g5; Nt5dc2; Hyal1; Fam213b; Phgdh; Hmgcs2; Csad; Smpd2; Lcn2; Plcb4; Inpp5f; C1galt1; Ndufa10l1; Upp1; Inpp4b; Ndst4; Ireb2;Gnpda2; Idi1; Pik3c2a; B3galt1; Rev3l; Pgap1; Ptgs2; Gad2; Csgalnact1; Dgkb; Nmnat2.
ko04144: Endocytosis	Folr1; Ntrk1; RT1-CE10; RT1-CE7; Rab11fip1; RT1-A3; RT1-A1; RT1-A2; Met LOC360231; RT1-T24-4; RT1-CE16; Rt1.aa; Hspa1a; RT1-T24-3; Hook1; Ofd1; Cav2; Ret; Txlng; Tmf1; Dnm1l; Filip1; Golga1; Wwp1.
ko05166: HTLV-I infection	Mybl2; Cd3e; RT1-CE10; Msx1; RT1-CE7; RT1-A3; RT1-A1; RT1-A2; LOC360231; RT1-T24-4; RT1-CE16; Rt1.aa; Wnt3; Fos; Atf3; RT1-T24-3;Wnt9b; Adcy8; Zfp36; Creb1; Arhgap29; Crem; Atf2; Fzd3; Mapk8; Myc.
ko05200: Pathways in cancer	Ntrk1; Gstm4; Mmp2; Wnt3; Fos; Tcf7l2; Mmp9; Kitlg; Wnt9b; Brca2; Vegfa; Ccne2; Arhgap29; Cpne9; Met; Ret; Hsp90aa1; Lef1; Rassf5; Fzd3; Hif1a; Stat3; Mapk8; Myc; Ptgs2.
ko04514: Cell adhesion molecules	Glycam1; Cldn2; Cdh3; RT1-CE10; Selplg; RT1-CE7; Cldn1; RT1-A3; F11r; RT1-A1; RT1-A2; LOC360231; RT1-T24-4; RT1-CE16; Rt1.aa; PVR; RT1-T24-3; Ncam2; Negr1; Cpne9; Nlgn1; Cadm1; Nrxn1.
ko04010: MAPK signalling pathway	Pla2g5; Ntrk1; Hspa1a; Fos; Hspb1; Map3k2; Dusp1; Cacnb4; Cacna2d1; Gadd45a; Arhgap29; Cpne9; Gadd45g; Taok1; Atf2; Ppm1l; Rasa1; Mapk8; Bdnf; Myc; Prkacb.
ko04145: Phagosome	RT1-CE10; RT1-CE7; RT1-A3; RT1-A1; Fcgr3a; RT1-A2; LOC360231; RT1-T24-4; RT1-CE16; Rt1.aa; Tubb6; RT1-T24-3; Hook1; Dync2h1; Ofd1; Tmf1; Filip1; Golga1; Eea1; Crispld2; Nos1.
ko04530: Tight junction	Cldn2; Cldn1; F11r; Prkcd; Synpo2; Spta1; Cpne9; Phf20l1; Bicd1; Txlng; Tmf1; Smtn; Filip1; Rb1cc1; Golga1; Cttnbp2nl; Ccdc39; Cep83; Ppfibp1.
ko04510: Focal adhesion	Nexn; Shc4; Vegfa; Akap12; Pak3; Rock1; Arhgap29; Cpne9; Met; Cav2; Smtn; Shc3; Pdlim4; Mapk8; Ppfibp1; Arhgap5; Kdr; Arid4a.
ko04310: Wnt signalling pathway	Sfrp1; Wnt3; Plcb4; Tcf7l2; Wnt9b; Grem2; Camk2d; Rock1; Cpne9; Lef1; Fzd3; Fhod3; Mapk8; Ppfibp1; Myc; Prkacb.
ko04020: Calcium signalling pathway	Calml4; Htr5b; Nexn; Plcb4; Adra1b; Adcy8; Camk2d; Akap12; Tacr1; Atp2b4; Cpne9; Grm5; Cd38; Prkacb; Nos1; Arid4a.
Dopaminergic synapse	Calml4; Fos; Plcb4; Creb1; Scn1a; Camk2d; Arhgap29; Cpne9; Grin2b; Kif5b; Atf2; Kcnj3; Gria4; Mapk8; Clock; Prkacb.
ko04062: Chemokine signalling pathway	Cxcl13; Ccl2; Prkcd; Plcb4; Adcy8; Shc4; Rock1; Arhgap29; Cpne9; Shc3; Stat3; Jak2; Pdlim4; Ppfibp1; Prkacb.
ko04912: GnRH signalling pathway	Pla2g5; Calml4; Mmp2; Mmp14; Prkcd; Plcb4; Map3k2; Adcy8; Camk2d; Hbegf; Cpne9; Mapk8; Prkacb.
ko04728: Neurotrophin signalling pathway	Ntrk1; Tp73; Calml4; Prkcd; Shc4; Camk2d; Arhgap29; Shc3; Mapk8; Bdnf.

### Gene interaction network analysis of DEGs

**Figure 8:**
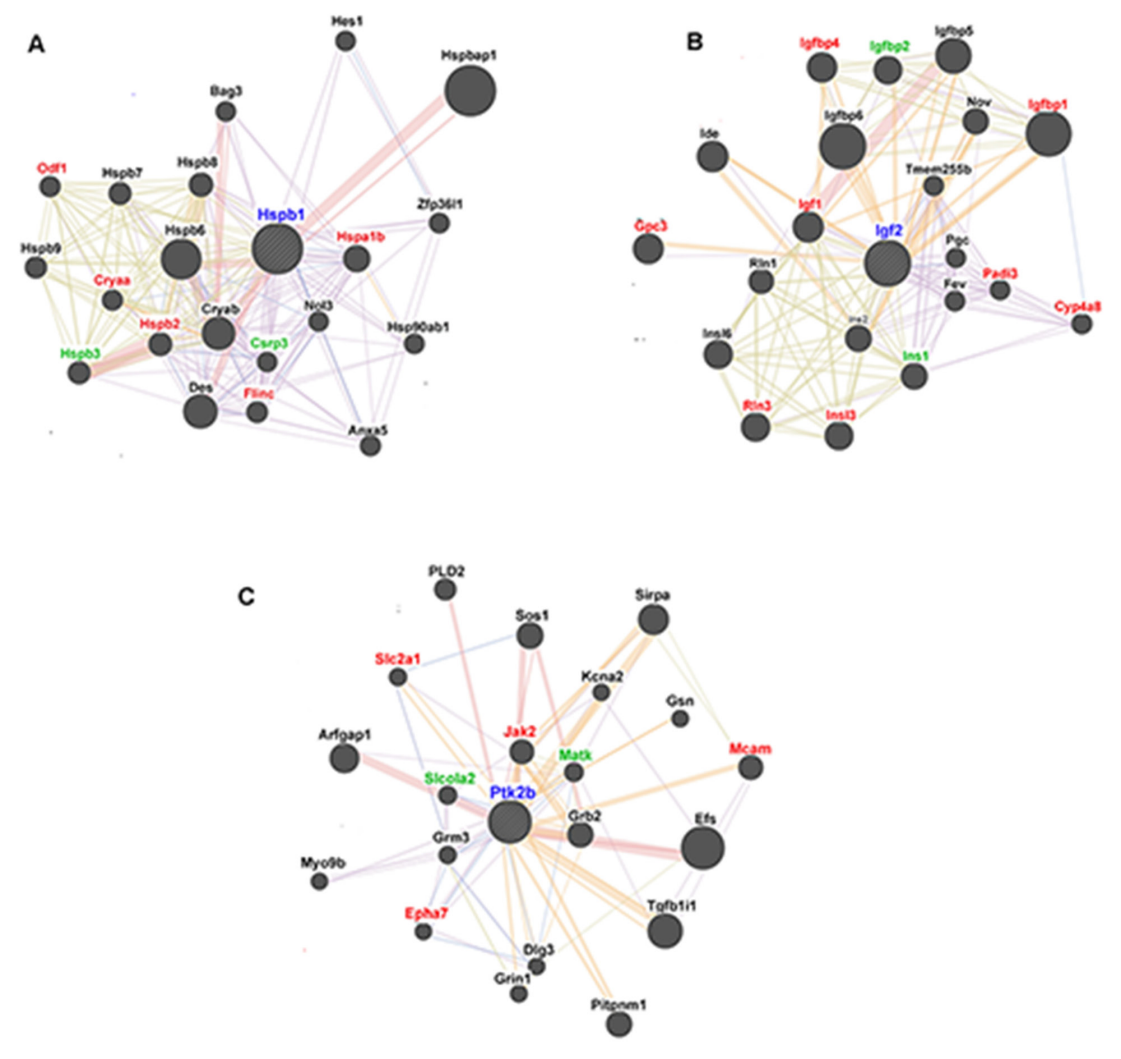
GeneMANIA functional interaction network analysis of candidate rat genes related to the neuroprotective effects of Orexin-A **(A)** Functional interaction network of Hspb1. **(B)** Functional interaction network of Igf2. **(C)** Functional interaction network of Ptk2b. Red symbols represent genes down-regulated in the Orexin-A group compared with the I/R group. Green symbols represent up-regulated genes. Black symbols indicate genes with no significant differences in expression between groups.

We selected three of the more interesting DEGs in the Orexin-A group compared with the I/R group for gene interaction analysis by GeneMANIA. The functional interaction network of Hspb1 (Figure 8A) includes seven Hspb1-interacting genes that were found to be differentially expressed by RNA-seq, of which five (Hspa1b, Odf1, Cryaa, Hspb2 and Flinc) were down-regulated and two (Hspb3 and Csrp3) were up-regulated (Table 5). Meanwhile, eight down-regulated and two up-regulated genes (Igf1, Igfbp1, Igfbp4, Gpc3, Padi3, Cyp4a8, Rln3, Insl3, Igfbp2 and Ins1; Table 5) appeared to functionally interact with Igf2 (Figure 8B). Similarly, six genes interacted with Ptk2b, of which Slc2a1, Jak2, Mcam and Epha7 were down-regulated and Slcola2 and Matk were up-regulated after reperfusion Orexin-A (Figure 8C and Table 5).

**Table 5:** Genes interacting with Hspb1, Igf2 and Ptk2b

**Genes interacting with Hspb1**					
**Gene symbol**	**Log2 ratio**	**Gene symbol**	**Log2 ratio**	**Gene symbol**	**Log2 ratio**
Hspb2	-9.95	Hspa1b	-1.51	Hspb3	8.45
Cryaa	-8.87	Flnc	-0.60	Csrp3	8.28
Odf1	-8.08				
**Genes interacting with Igf2**					
**Gene symbol**	**Log2 ratio**	**Gene symbol**	**Log2 ratio**	**Gene symbol**	**Log2 ratio**
Rln3	-10.19	Cyp4a8	-1.57	Igfbp2	1.75
Insl3	-8.81	Igf1	-1.16	Ins1	0.97
Igfbp1	-7.41	Igfbp4	-1.05		
Padi3	-6.32	Gpc3	-0.75		
**Genes interacting with Ptk2b**					
**Gene symbol**	**Log2 ratio**	**Gene symbol**	**Log2 ratio**	**Gene symbol**	**Log2 ratio**
Jak2	-1.13	Slc2a1	-0.56	Slcola2	0.75
Epha7	-0.69	Mcam	-0.53	Matk	0.71

In addition, we analysed the signalling pathways involving Hspb1, Igf2 and Ptk2b that may be involved in Orexin-A reperfusion. RNA-seq data showed that Hspb1 was mainly involved in the MAPK and VEGF signalling pathways, whereas Igf2 was most closely associated with the P53 signalling pathway. Ptk2b could potentially affect four signalling pathways: the chemokine, nicotine addiction, calcium and Jak-STAT signalling pathways. Elucidation of these DEGs and their associated signalling pathways could prove critical for understanding the neuroprotective mechanisms of Orexin-A.

## DISCUSSION

RNA-seq technology has emerged as a powerful method for screening transcripts and has been applied to a broad range of fields [18, 19]. Bermudez et al. used RNA-seq to screen DEGs and corresponding pathways involved in glucocorticoid responsiveness in a bovine trabecular meshwork (BTM) cell strain. Sequencing results showed that 93 and 606 genes were differentially expressed in responder and non-responder BTM cells, respectively [20]. Wang et al. analysed stage-specific genes associated with gastric cancer using RNA-seq and identified 2,224 genes specifically expressed in stages I and II, and 539 genes specific to stage IV [21]. Meanwhile, Holmes et al. assessed the impact of three different intensities of transcranial direct current stimulation (tDCS) on gene expression in rat cerebral cortex by RNA-seq. Compared with the sham group, ~1,000 DEGs were identified at each treatment intensity [22]. In the current study, Orexin-A alleviated the cerebral infarction volume induced by I/R injury, consistent with the neuroprotective effects of Orexin-A on I/R injury. To more comprehensively reveal which genes and pathways are involved in the neuroprotective effects of Orexin-A, RNA-seq was carried out on sham, I/R and Orexin-A rat groups. To our knowledge, this is the first study to use RNA-seq to perform global gene expression profiling after reperfusion with Orexin-A in the hippocampus. Compared with the I/R group, ≥2-fold up-regulation of 149 genes and ≥2-fold down-regulation of 500 genes was observed following reperfusion with Orexin-A, identifying 649 genes that are potentially involved in the neuroprotective effects of Orexin-A.

Go analysis based on the RNA-seq data identified DEGs involved in adhesion, metabolism, localisation, immune reaction, programmed cell death and defence responses (Figures 5 and 6; Table 3). We screened several immune response-related genes including IL11 (-2.359), Cd55/44 (-1.681/-3.4947), Ccl2 (-4.059), Ifit1 (-4.2288) and Cadm1 (-1.079) differentially expressed after reperfusion with Orexin-A. A number of genes related to cell death, including Bcl3 (-4.9307), caspase 3/4 (-0.826/-1.838), Rassf5 (-1.2599), Myc (-1.0524), Casp8ap2 (-1.307) and Bag4 (-1.0895), were down-regulated in the Orexin-A group. In addition, reperfusion induced the up-regulation of Hif1a, Stat3, Hspb2, Hspa1a, Hspa1b and Hspb1, which play an important role in stimulus or stress, but Orexin-A lowered their expression levels (log2 ratio) by -1.1357-, -1.135-, -9.951-, -3.474-, -1.51- and -2.602-fold, respectively. These differences in expression after reperfusion with Orexin-A may contribute to its neuroprotective effects.

Orexin-A has been shown to activate multiple protein kinases such as PKA, PKC, MAPK/Erk and PDK1 in various cellular contexts [23]. Orexin-A activates the mTORC1 pathway via extracellular calcium influx and the lysosome pathway in recombinant cell lines, leading to the central regulation of cell growth and metabolism [24]. Orexin-A increases glucose transporter 4 expression and lipid accumulation in 3T3-L1 adipocytes via ERK1/2, JNK and p38 MAPK signalling [25, 26]. Orexin-A treatment promoted the uptake of Glu via the OX1R/PKCα/ERK1/2 pathway and protected co-cultured cells against anoxia/hypoglycaemic injury in astrocytes [27]. Orexin-A stimulates ERK_1/2_ phosphorylation and facilitates cell migration via the PLC-PKCα signalling pathway in cultured rat astrocytes [28]. In the current study, KEGG pathway analysis identified 206 pathways that were closely associated with Orexin-A reperfusion, of which metabolic pathways accounted for 34 DEGs, and MAPK signalling accounted for 21 DEGs. It was not surprising to find that metabolic pathways and MAPK signalling pathways may play important roles in Orexin-A reperfusion.

Besides these two pathways, several others were also detected, including Wnt, calcium, chemokine, gonadotropin-releasing hormone (GnRH) and neurotrophin signalling pathways. Orexins play important roles in nociceptive modulation via activating intracellular calcium signalling [29]. Orexin-A and Orexin-B significantly increase the expression of neurotrophin-3 (NT-3) at the mRNA level when primary cortical neurons are cultured with 0.01, 0.1 or 1 mM Orexins [30], and Orexin-A directly stimulates GnRH neurons to secrete GnRH, implicating Orexin-A in GnRH signalling [31]. Taken together, these results implied that Orexin-A might regulate the above signalling pathways, and thereby modulate cerebral I/R injury.

Among the identified DEGs, three genes of particular interest were selected for analysis using GeneMANIA. Heat shock protein beta-1 (Hspb1), also known as Hsp27, is a member of the small HSP family and a key mediator in the cellular response to environmental and physiological stresses [32–34]. Upon expose to oxidative stress, HSP27 functions as an antioxidant to decrease the levels of reactive oxygen species (ROS) [35]. In benign prostatic hyperplasia (BPH), the expression of HSP27 is gradually increased and accompanied by inflammation [36]. In retinal capillary endothelial cells, HSP27 functions as an anti-apoptotic protein to preserve the endothelial barrier [37]. Others have similarly noted that HSP27 inhibits cell death via a c-jun-mediated cascade in cerebral ischemia [38]. HSP27 transgenic mice display amelioration of the infarction volume, behavioural deficits and blood brain barrier (BBB) damage in a stroke model [39]. Insulin-like growth factor 2 (Igf2) is indispensable in a number of cells because it promotes cellular proliferation and differentiation [40]. In medulloblastoma, Igf2 promotes the proliferation of precursor cells and the PZp53 cell line [41]. IGF2 also mediates the proliferation of hippocampal neural stem cells via IGF1R and AKT-dependent signalling [42]. IGF2 is highly expressed in microglia and exhibits a strong protective effect against cytokine-mediated neuronal death *in vitro* and *in vivo* [43]. Protein tyrosine kinase type 2 beta (Ptk2b) was found to play a vital role in the response to diverse cellular stimuli and diseases. Ptk2b specifically induces cell death and reduces clonogenic growth in myeloma cell lines and multiple myeloma stem cells. Furthermore, Ptk2b inhibition attenuates multiple myeloma progression [44]. Ptk2b can be up-regulated by lovastatin, resulting in an anti-apoptotic effect on ox-LDL-induced cell injury in endothelial cells. The protective effect of Ptk2b is associated with the AKT signalling pathway [45]. At 24-72 h after focal cerebral ischemia, phospho-Ptk2b was rapidly induced in microglia surrounding the necrotic infarction area, and phospho-Ptk2b was confirmed to colocalise with phospho-p38 and act as an upstream mediator of the p38 signalling pathway after cerebral ischemia [46]. Consistent with these previous findings, these three genes likely play a vital role in Orexin-A-mediated neuroprotection, and their functions and molecular mechanisms will be investigated in future studies.

It has found that orexin-A has multiple effects on different tissues. By comparing the mechanism of orexin-A in different tissues, it can be found that there is commonality and tissue specificity. It will provide a comprehensive understanding of Orexin-A effects on different organs and reveals whether its neuroprotective effects are tissue specific at molecular levels.

In conclusion, our global expression profiling identified genes, pathways, functions and interaction networks potentially involved in the neuroprotective effects of Orexin-A reperfusion. However, the results obtained by RNA-seq and bioinformatic analysis were general and indicate the involvement of numerous complex processes, and further experiments are needed to validate the identified DEGs and signalling pathways to clarify the molecular mechanisms involved in the process of Orexin-A reperfusion.

## MATERIALS AND METHODS

### Animals

Male Wistar rats, aged 6-8 weeks, were obtained from Pengyue Experimental Animal Breeding Institute of Jinan, China (License No. SCXK (Lu) 2014-0007). All rats were maintained in a room at 25 ± 3°C and 50-60% humidity, with a 12 h light-dark cycle and free access to food and water. All experiments were approved by the Ethics Committee of Jining Medical University and performed in accordance with the National Health Guide for the Care and Use of Laboratory Animals.

### Animal grouping

A total of 18 rats were randomly divided into three groups: (1) sham-treated group (sham), from which the right carotid artery was isolated without further processing; (2) I/R group, in which rats were subjected to a 2 h right middle cerebral artery occlusion (MCAO) followed by a 24 h reperfusion with saline (10 μl, 0.9% NaCl) after ischemia at the onset of reperfusion; (3) Orexin-A reperfusion group (Orexin-A), in which rats were injected with Orexin-A (50 ng/kg; Phoenix Pharmaceuticals, Inc., Burlingame, CA, USA) after reperfusion.

### MCAO model

Rat MCAO model experiments were performed using the bolt line method. Firstly, rats were anesthetised by intraperitoneal injection of 10% chloral hydrate (0.3 mL/100 g) and swabbed with iodophor. The common carotid artery (CCA) was isolated, and a midline incision was made to expose the internal carotid artery (ICA), external carotid artery (ECA) and right CCA. A nylon suture (0.26 mm diameter) was inserted into the right CCA lumen and gently injected into the ICA at a depth of 18 mm, and the neck incision was sutured. Finally, nylon sutures were gently removed at 2 h after ischemia, and reperfusion was performed for 24 h.

### TTC staining

The cerebral infarction volume was measured using TTC (Sigma, St Louis, MO, USA) staining. At 24 h after reperfusion, brains were removed, frozen at -20°C for 30 min, cut into 2 mm thick coronal slices and stained with 0.2% TTC solution in the dark for 30 min at 37°C. After fixing with 4% formalin, slices were photographed using a IX71 microscope (Olympus, Japan). Infarction zones were analysed with Image-Pro Plus v 6.0 software (Media Cybernetics, Bethesda, MD, USA). The infarction vs. normal volume percentage was calculated.

### Construction of cDNA library and RNA-seq

Total RNA was extracted using TRIzol reagent (Invitrogen, Carlsbad, CA, USA) according to the manufacturer's instructions. RNA was quantified and the integrity was determined using a BioSpec-nano spectrophotometer (Shimadzu, Japan). Total RNA was treated with DNase I to remove DNA contamination, and RNA was used to generate a cDNA library. The mRNAs were multiplexed and tagged with standard Illumina tags, and fragments were amplified by PCR. Library quality and quantity were determined using an Agilent 2100 Bioanalyzer and an ABI StepOnePlus Real-Time PCR System. Library products were sequenced using an Illumina HiSeq TM 2000 platform by Shenzhen Huada Genetics Corporation (Shenzhen, China).

### Bioinformatics analysis

Raw read data from RNA-seq were subjected to quality control (QC) that involved discarding low-quality reads, and the resulting clean reads were aligned with reference sequences using BWA software [47]. Expression levels of genes were quantified using RSEM [46]. Screening of DEGs was based on three methods: Poisson distribution, the NOISeq package [48] and the EBSeq package [49]. Gene Ontology (GO; http://www.geneontology.org/), PANTHER classification analysis (http://www.pantherdb.org/), KEGG Pathway enrichment analysis (http://www.genome.jp/kegg/) and protein-protein interaction network analysis (GeneMANIA; http://genemania.org/) were subsequently performed.

### qRT-PCR

The same RNA samples were reserve-transcribed into cDNA using a cDNA synthesis kit (Tiangen Biotech, Beijing, China) according to the manufacturer's protocol. qRT-PCR was performed in 96-well plates using a Roche LC480. Reactions (20 μL) were mixed with 10 μL of 2×SYBR SuperReal PreMix (Tiangen Biotech, Beijing, China), 2 μL of cDNA, 0.6 μL of forward primer, 0.6 μL of reverse primer and 6.8 μL of ddH_2_O. The amplification procedure was as follows: 95°C for 10 min, followed by 40 cycles of 95°C for 15 s and 60°C for 1 min. PCR primers are listed in Table 1. β-actin was amplified as an endogenous control. The relative mRNA level of each gene was calculated using the 2 ^−ΔΔCT^ method. At least three replicates were performed for each cDNA sample, and reactions were prepared in triplicate.

### Statistical analysis

All data are expressed as mean ± standard error of the mean (SEM) and were analysed using GraphPad Prism one-way analysis of variance (ANOVA). A *p*-value less than 0.05 was considered statistically significant.
